# Charlotte Medical Journal

**Published:** 1892-07

**Authors:** 


It is our pleasure to note the advent of a new-comer into medical journalism, the *Charlotte Medical Journal*, which is published in Charlotte, N. C., by Drs. E. C. Register and J. C. Montgomery. The first issue shows with what careful precision it has been edited, and its appearance is altogether quite attractive. We predict for it a bright career.

